# Ameliorative effects of supranutritional selenium on TLR‐4‐NF‐kB‐TNF‐α‐mediated hepatic oxidative injury and inflammation in goats fed high concentrate diet

**DOI:** 10.1002/fsn3.2980

**Published:** 2022-07-18

**Authors:** Tahmina Shah, Moolchand Malhi, Allah Bux Kachiwal, Bachal Bhutto, Qurban Ali Shah, Yan Lei, Saeed Ahmed Soomro, Jamila Soomro, Nazeer Hussain Kalhoro, Hongbing Gui

**Affiliations:** ^1^ Department Veterinary Physiology and Biochemistry Sindh Agricultural University Tandojam Pakistan; ^2^ Department of Veterinary Parasitology Sindh Agricultural University Tandojam Pakistan; ^3^ Department Veterinary Pathology Lasbela University of Agriculture, Water and Marine Science Uthal Balochistan Pakistan; ^4^ Dairy Herd Improvement Center Henan Animal Husbandry Bureau Zhengzhou China; ^5^ Sindh Institute of Animal Health Karachi Pakistan; ^6^ Institute of Animal Sciences Jiangsu Academy of Agriculture Science Nanjing China

**Keywords:** acute phase response, cytokines, interleukins, lipopolysaccharides, oxidative stress

## Abstract

We examined whether surplus dietary selenium (Se) supply could alleviate high concentrate (HC) diet‐induced hepatic oxidative stress (OS) and inflammation. Eighteen young goats were distributed into three groups; were fed low (LC, concentrate: forage; 35: 65), high concentrate (HC, 65: 35), or Se‐supplemented HC (HCSe, 65: 35 + 0.5 mg Se kg^−1^ diet) diets for 10 weeks. Short chain fatty acids, OS markers and immunoinflammatory genes expressions were assessed through gas chromatograph, kits, and RT‐qPCR, respectively. Compared with LC, HC diet increased (*p* < .05) colonic and serum lipopolysaccharide (LPS) levels and induced hepatic oxidative injury by increasing (*p* < .05) malondialdehyde (MDA) levels and decreasing (*p* < .05) activities of glutathione peroxidase, superoxide dismutase, and catalase. HC diet altered hepatic mRNA expressions of toll‐like receptor‐4 (TLR‐4), cluster of differentiation‐14 (CD‐14), tumor necrosis factor‐α (TNF‐α), TNF receptor‐associated factor‐6 (TRAF‐6), nuclear factor kappa B (NF‐κB), interleukin‐1β (IL‐1β), IL‐10, IL‐13, LPS‐binding protein (LBP), serum amyloid A (SAA), α‐acid glycoprotein (AGP), and albumin (ALB). Conversely, extra‐Se supply lowered LPS and attenuated antioxidant status and inflammation in liver. In conclusion, HC diet induced oxidative lesions and TLR‐4 pathway‐mediated inflammation, whereas supranutritional Se alleviated oxidative and inflammatory lesions through TLR‐4 pathway regulation in goat liver.

## Graphical abstract

HC diet increased colonic and serum lipopolysaccharide levels and induced hepatic oxidative injury and inflammation by altering activities of oxidative stress related enzymes and expression of immuno‐inflammatory genes including cytokines, acute phase proteins (APPs) and receptor proteins. Conversely, supranutritional Se alleviated oxidative and inflammatory lesions through TLR‐4‐NF‐kB‐TNF‐ pathway regulation in goat liver.

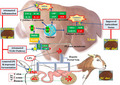

## 1 INTRODUCTION

Ruminants are fed high concentrate (HC) diets particularly during growth, fattening and productive phases in their life‐cycles to hasten growth and production performances (Hua et al., 2017; Plaizier et al., 2017). However, prolonged HC feeding causes gastrointestinal tract (GIT) disorders leading to complex systemic complications and eventually raises the concerns over economy, animal welfare, and environment (Hua et al., 2017; Zhang, Liu, et al., 2019, Zhang, Meng, et al., 2019). The principal reason of GIT disturbances is the dysfermentation associated with multiple sequential events including microbiota dysbiosis, short chain fatty acids (SCFA) accumulation due to too rapid production to absorb with simultaneous lowered luminal pH in rumen and hindgut (Li et al., 2012; Tao et al., 2017). Consequently, the acidic luminal environment provokes a considerable release of endotoxins particularly lipopolysaccharides (LPS) thereby induce mucosal injuries and alter permeability in GIT wall including rumen, colon and caecum, and ultimately lead to LPS translocation (Abaker et al., 2017; Li et al., 2012). Although, the LPS can be translocated from both rumen and hindgut (colon and cecum) wall but the hindgut epithelium, unlike rumen epithelium, being monolayered and lacking natural buffering protection from overacidity, is more susceptible to abnormal luminal conditions thus believed to be major site for LPS translocation (Li et al., 2012; Tao et al., 2017).

Liver is an important homeostatic organ which perform several important functions including defense against microbes and toxins (Chang, Zhang, Xu, Jin, Guo, et al., 2015). Once leaked from GIT epithelium, via hepatic portal vein (PV), LPS reaches into the liver, where the exceeding LPS levels impair the hepatocytes and eventually escape through hepatic vein (HV) in peripheral circulation (Chang, Zhang, Xu, Jin, Guo, et al., 2015; Dong et al., 2013). Feeding HC diets have been shown to decrease hepatic LPS removal rate and increase blood LPS levels accompanied with histopathological lesions in liver (Chang, Zhang, Xu, Jin, Guo, et al., 2015; Dong et al., 2013; Guo et al., 2017). Moreover, LPS‐induced histopathological lesions were associated with oxidative stress (OS) and inflammation (Duanmu et al., 2016). On interaction with hepatocytes, the LPS stimulate innate immunity through acute phase proteins (APPs) modulation; thus, the resultant fluctuation in blood APPs concentrations reflects the functional disturbance and inflammation of liver (Guo et al., 2017; Minuti et al., 2015). APPs including serum amyloid A (SAA), α‐acid glycoprotein (AGP), LPS‐binding protein (LBP), haptoglobin (Hp), and albumin (ALB) have been found to be modulated by HC feeding (Chishti et al., 2020; Li et al., 2019; Ohtaki et al., 2020). LBP is highly sensitive to LPS, and its plasma levels drastically raise up to 200% in goats fed HC diets and hence considered as reliable biomarker of systemic inflammation (Chang, Zhang, Xu, Jin, Seyfert, et al., 2015; Dong et al., 2013). The APPs production is stimulated by HC diet‐derived LPS in liver through activation of toll‐like receptor‐4 (TLR‐4)‐mediated nuclear factor kappa B (NF‐kB)‐tumor necrosis factor‐α (TNF‐α) signaling pathway in immune cells (Ciesielska et al., 2021; Kany et al., 2019). It has been shown that HC diets induce NF‐κB expression through LPS and thereby modulate the expressions of related cytokines, such as TNF‐α, interleukin‐1β (IL‐1β), IL‐6, and IL‐10, and consequently altered the AAPs production in livers of ruminants (Chang, Zhang, Xu, Jin, Guo, et al., 2015; Dong et al., 2013; Guo et al., 2017).

Selenium (Se) is an essential dietary micronutrient, known for its multiple biochemically important actions (He et al. 2021; Sherlock et al., 2020; Ahmed et al., 2016). Its antioxidant effects are related to a class of Se‐containing enzymes, glutathione peroxidases (GSH‐Px) which are generally found in all tissues, being proportionally higher in Se‐specific tissues like liver (Bano et al., 2019; Čobanová et al., 2017; Shahid et al., 2020). In addition, the bioavailability and thus production and release of GSH‐Px depend upon the form and concentration of Se in diet and the nature of diet. The flow of Se down the GIT and its assimilation in body tissues was greater in animals fed concentrate diets than those fed roughage‐based diets (Hernández‐Calva et al., 2007; Samo et al., 2018). Moreover, the animals given surplus Se (20–30 times over the optimal levels) in normal conditions retained more Se with higher antioxidant stability in tissues without compromising animal health (Del‐Razo‐Rodriguez et al., 2013). In particular, the dietary extra‐Se addition in stressful conditions have been shown to exert protective effects. The surplus Se levels ranging from 0.6 to 0.7 mg kg^−1^ diet mitigated concentrate diet‐induced oxidative injury and apoptosis in colonic epithelium of goat (Samo et al., 2020) and reduced stress during and after calving in cattle by enhancing immune response through attenuation of APPs, including SAA, Hp, and albumin (Gong & Xiao, 2021; Hall et al., 2014). The oversupply of Se in feed increased blood antioxidant status and improved immune health in sheep and pigs under heat stress (Chen et al., 2020; Liu, Chen, et al., 2021) and alleviated metabolic impairment by recovering the gene expression profile in swine liver exposed to chronic heat stress (Liu, Tang, et al., 2021). Attenuating effects of Se against hepatic OS and inflammation in heat‐stressed and drug‐stressed growing pigs (He et al. 2021; Liu, Tang, et al., 2021), and in LPS‐challenged chicken occurred through regulation of immunoinflammatory genes including receptor proteins, transcriptional factors, adaptor proteins, and AAPs involved in TLR‐4 signal transduction pathway (Qu et al., 2020).

Furthermore, the OS and inflammatory responses in liver of ruminants fed HC diets are suggested to be mediated by GIT‐derived LPS (Chang, Zhang, Xu, Jin, Guo, et al., 2015; Guo et al., 2017). Since, LPS‐challenge causes hepatic oxidative injury leading to inflammation, along with lowered Se levels and GSH‐Px activities in liver and serum, and impaired hepatic selenoproteins metabolism in mice, sheep, and goats (Cao et al., 2006; Sherlock et al., 2020; Wang et al., 2017). This emphasizes the importance of Se in LPS‐induced inflammatory response and indicates an excessive utilization of selenoproteins and thus increasing demand of Se in stressful conditions. These data led us to hypothesize that extra‐Se feeding would attenuate the concentrate diet‐induced hepatic damage by enhancing the antioxidative stability and impeding the inflammatory pathway in liver. Thus, the current study evaluated the effects of Se‐oversupply through organic source against HC diet‐induced oxidative stress and inflammatory status in the liver of goats.

## 2 MATERIALS AND METHODS

### 2.1 Animals, adaptation, and feeding management

The experiments performed in these studies were approved by the Institutional Ethical Committee, Sindh Agriculture University Tandojam. The present research used a total of 18 young goats, about 3.5 months old and 12 kg body weight (BW). Goats were placed individually in a cage with an area (Sq. ft) of 2.5 × 4. Animals were divided into three dietary groups; and fed low concentrate (LC, concentrate: forage; 35: 65), high concentrate (HC, 65: 35) or Se‐supplemented HC (HCSe, 65: 35 plus 0.5 mg Se kg^−1^ diet) diets, with 6 animals per group. Selenium was provided through organic Se i.e., Se‐yeast (Selemax, Biorigin, Sao Paulo, SP 18680‐9, Brazil). During adaptation period of 4 weeks, all goats were fed LC diets for first 2 weeks and then the concentrate levels were slowly raised to the proposed levels in HC and HCSe diets by the next 2 weeks. Meanwhile, Se levels were slowly raised to proposed levels in HCSe diet.

Animals were fed two times a day; at 08:00 h and 17:00 h for 10 weeks. Water was freely available. Original Se contents (mg kg^−1^ diet) in LC and HC feeds were 0.035 and 0.15, respectively. Se concentrations in LC diets were leveled with HC diets by adding 0.115 mg Se kg^−1^ diet. After addition of 0.5 mg Se kg^−1^ diet, HCSe group received a total of 0.65 mg of Se kg^−1^ diet. Chemical ingredients and Se concentrations in diets are given in Table 1. Se levels in diets were analyzed via inductively coupled plasma‐mass spectrometry (ICP_OES) Optima_2100 DV equipment (Perkin‐Elmer, USA) as reported by Taylor (2005).

**TABLE 1:** Composition and Se levels in diets fed to goats

Items	Treatments		
	LC	HC	HCSe
Ingredients (% of DM)			
Corn	25.6	25	25
Wheat bran	–	30.7	30.7
Soybean meal	7.4	2.2	2.2
Rapeseed meal	–	4	4
Limestone	0.5	1.5	1.5
DCP	0.8	0.7	0.7
Salt	0.4	0.4	0.4
Mineral Premix^a^	0.4	0.4	0.4
Se (mg kg^−1^ diet)			
Background Se in diet	0.035	0.15	0.15
Added	0.115	–	0.5
Total level	0.15	0.15	0.65

*Note*: Goats were fed low concentrate (LC), high concentrate (HC), and HC with selenium (HCSe) diets for 10 weeks. DM, Dry matter; DCP, Digestible crude protein.

^a^ Per kg of premix = Vitamin A 6000 U; Vitamin D2 500U; Vitamin E 80 mg; Cu 6.25 mg; Fe 62.5 mg; Zn 62.5 mg; Mn 50 mg; I 0.125 mg; Co 0.125 mg; Mo 0.125 mg. Selenium was supplemented as selenium yeast (SY) in powder form added to HC diet.

### 2.2 Slaughtering of animals and sample collection

Blood sample (10 ml) was collected from each animal just prior the slaughtering. Serum was acquired by centrifuging blood samples at 3000 ***g*** at 4°C for 15 min and stored frozen for future use. Goats were then euthanized, instantly, the abdomen was incised, and colon and liver were carefully isolated and removed. After aseptic collection of colonic contents (digesta) samples, the pH was quickly measured and then the samples were stored frozen for the determination of short chain fatty acids (SCFA). Liver was rinsed with cold PBS and then a tissue‐piece of adequate size was sliced from the center of the right lobe, a portion of which was cut into pieces (1 cm^2^) and preserved into 10% paraformaldehyde solution for histomorphological analysis. Another portion was preserved in Eppendorf tube at – 80°C until analysis for oxidative stress markers mRNA expression.

### 2.3 Liver histology

Hepatic tissue samples from the center of the right lobe of four goats in each group were utilized to assess the histopathology. After overnight fixation in 4% paraformaldehyde, the tissue samples were subjected to dehydration, clearing, and paraffin‐embedding as previously described by Moolchand et al. (2013). The sections were sliced at 4–6 μm thickness and subjected to the conventional hematoxylin and eosin (H&E) staining system. At least 10 replications were measured for each animal.

### 2.4 Samples analyses (Determinations of SCFA, LPS contents, and oxidative stress markers)

The digesta samples from colon of each goat were taken in equal amount of saline buffer, thoroughly mixed and then subjected to centrifugation at 3000 ***g*** for 15 min. The resultant supernatant was assembled into two parts for analysis of LPS contents and SCFA analysis, respectively. LPS contents in colonic digesta and serum samples were detected by chromogenic endpoint assaying‐kit (Xiamen Bioendo Tech. Co., Ltd. China). Digesta (pretreated) supernatants and serum samples were undergone dilution at the rate of 0.1–1.0 endotoxin units per ml and assessed. Another portion of the digesta supernatant was processed for SCFA analysis as illustrated by Malhi et al. (2013) using column (capillary and packed) gas chromatograph (GC 2014‐B; Shimadzu‐Corp., Tokyo‐Japan); Column dimensions: 30 m × 0.32 mm × 0.25 mm; temperatures (°C) of column, injector, and detector range up to: 110, 180, and 180, respectively. The hepatic tissues were homogenized in regular PBS buffer and then processed for the determination of contents of malondialdehyde (MDA) and enzymatic activities of glutathione peroxidase (GSH‐Px), superoxide dismutase (SOD), and catalase (CAT) using commercial kits from Jincheng Bio‐engineering institute (Nanjing Jincheng Techn. Co. Ltd. PR China). The instructions and protocols by manufacturers were strictly followed wherever the commercial kits were used for specific sample analysis.

### 2.5 Total RNA isolation and real‐time Rt‐PCR

Hepatic tissue samples were processed for total RNA extraction by means of TRIZOL reagent (Takara, Dalian, China) followed by subsequent quantification in a nano drop spectrophotometer and verification through ethidium bromide‐stained 1.4% agarose‐formaldehyde gels electrophoresis. Total RNA (2 μg) sample was processed for reverse transcription (RT) in a mixture containing reverse transcriptase (100 U), RNase inhibitor (8 U) (Promega, USA), random 6 bp primers (5.3 mmol L^−1^), dNTP (0.8 mmol L^−1^) (TaKaRa, Dalian, China), and finally 16RT‐buffer was added to make total volume of 25 ml and then incubated at 37°C for 1 h immediately after terminating the reaction at 95°C for 5 min, the RT product (cDNA) was ice‐cooled. The cDNA (2 μl) sample was subjected to Rt‐PCR in a final volume of 25 ml containing 12.5 ml PCR SuperMix (1xiQ SYBR Green, Bio‐Rad Lab., Hercules, CA) and 0.6–0.8 mM of primers for target genes (Table 2). After denaturation process at 95°C for 1 min., the sample was amplified under three‐step process (20 s at 95°C, 20–30 s at 60–64°C, 30 s at 72°C) and the process was repeated for 40–45 cycles. Relative mRNA expressions of genes were determined using formula 2^−△△Ct^, after normalization with Glyceraldehyde 3‐phosphate dehydrogenase (GAPDH).

**TABLE 2:** The size (bp), nucleotide sequence, and accession number of primers

Genes	Primer sequence (5′ → 3′)	Size (bp)	Accession no.
TLR4	**F:** GTTTCCACAAGAGCCGTAA **R:** TGTTCAGAAGGCGATAGAGT	195	JQ342090.1
CD14	**F:** CCGTTCAGTGTATGGTTGCC **R:** TGCTTCGGGTCGGTGTT	239	NM_001077209.1
MyD88	**F:** ACAAGCCAATGAAGAAAGAG **R:** GAGGCGAGTCCAGAACC	98	98 JQ308783.1
TRAF6	**F:** GCGGCCTTCAAGTTAGGAGA **R:** TCATCAACTGCTCGTTCGGG	141	NM_001034661.2
NF‐κ B	**F:** ACGATCGTCACCGGATTGAG **R:** GGTGCTGAGAGATGGCGTAA	194	XM_005699996.1
TNF‐α	**F:** CAAGTAACAAGCCGGTAGCCC **R:** CCTGAAGAGGACCTGCGAGTAG	173	AF276985.1
IL‐1β	**F:** GAAGAGCTGCACCCAACA **R:** CAGGTCATCATCACGGAAG	172	D63351.1
IL‐6	**F:** GGAGGAAAAGGACGGATGCT **R:** GGTCAGTGTTTGTGGCTGGA	226	EU276071.1
IL‐10	**F:** TTAAGGGTTACCTGGGTTGC **R:** CCCTCTCTTGGAGCATATTGA	178	DQ837159.1
IL‐13	**F:** CAGTGTCATCCAAAGGACCAAG **R:** CGGACGTACTCACTGGAAACC	248	NM_174089
LBP	**F:** CAAGTAACAAGCCGGTAGCCC **R:** CCTGAAGAGGACCTGCGAGTAG	138	XM_004014566.1
SAA	**F:** CATCCTGCGTCTGGACCTGG **R:** TTCCTTGATGTCACGGACGATTT	121	AF540564.1
Hp	**F:** TAATGCCCATCTGCCTAC **R:** CGCCCTCATAGTGTTTCA	162	XM_004015111.1
AGP	**F:** TTGCTTGGCTGCAGGTGT **R:** CAATGGTCTGGTACTCTCTCTG	197	XM_012152252
ALB	**F:** TAGCTCGCCTGAGCCAGAAA **R:** GCAAGATCTGCCCTGTCGTC	136	NM_001009376.1
GAPDH	**F:** GGGTCATCATCTCTGCACCT **R:** GGTCATAAGTCCCTCCACGA	180	HM043737.1

*Note*: Toll‐like receptor (TLR)‐4, Cluster of differentiation (CD)‐14, Myeloid differentiation (MyDD)‐88, Tumor necrosis factor (TNF) ‐α, TNF receptor‐associated factor (TRAF)‐6, Nuclear factor kappa light chain enhancer of activated B cells (NF‐κB), Interluekins (IL)‐1β, IL‐6, IL‐10, IL‐13, Lipopolysachharide‐binding protein (LBP), Serum amyloid A (SAA), α‐Acid glycoprotein (AGP), Haptoglobin (Hp), Albumin (ALB), and Glyceraldehyde 3‐phosphate dehydrogenase (GAPDH).

### 2.6 Statistical analysis

Data between three dietary groups were compared by one‐way analysis of variance (ANOVA) using SPSS 16.0 (Stata_Soft, Tulsa‐OK). Following observation of significant *F‐*value, the significance between groups was revealed through Tukey's post hoc test application. Data are stated as the means ± standard error of means (SEM). Significant differences were estimated at *P*‐value below 0.05.

## 3 RESULTS

### 3.1 Colonic fermentation pattern

The molar concentrations of acetate (Ac), propionate (Pr), and total short chain fatty acids (tSCFA) in colonic fluid significantly enhanced (*p* < .05) and the pH reduced (*p* < .05) in HC and HCSe groups compared with LC group (Table 3). However, butyrate (Bu) concentration and Ac: Pr ratio revealed significant increase (*p* < .05) in HC than in LC, but no difference (*p* > .05) was observed between LC and HCSe treatments. Moreover, the concentrations of tSCFA, Ac, and Ac: Pr ratio were lowered (*p* < .05) by 9.9%, 17.1%, and 14.14% and the pH raised (*p* < .05) by 0.422 units in HCse compared with HC goats.

**TABLE 3:** The effect of LC, HC, and HCSe diets on SCFA concentration and pH in colonic fluid of goat

Items	Treatments		
	LC	HC	HCSe
Acetate (mmol)	27.21 ± 0.77^c^	40.60 ± 0.79^a^	33.66 ± 0.66^b^
Propionate (mmol)	15.86 ± 0.48^b^	18.56 ± 0.57^a^	20.02 ± 0.54^a^
Butyrate (mmol)	7.28 ± 0.21^b^	10.36 ± 0.30^a^	8.93 ± 0.41^ab^
tSCFA (mmol)	50.35 ± 1.07^c^	69.51 ± 1.28^a^	62.61 ± 1.22^b^
Ac: Pr Ratio	1.72 ± 0.07^b^	2.07 ± 0.073^a^	1.78 ± 0.076^b^
pH	6.58 ± 0.073^a^	5.83 ± 0.06^b^	6.25 ± 0.064^c^

*Note*: Ac:Pr, acetate to propionate ratio; tSCFA, total short chain fatty acid. Goats were fed low concentrate (LC), high concentrate (HC), and HC plus selenium (HCSe) diets for a period of 10 weeks. Total Se concentrations in LC, HC, and HCSe diets were 0.15, 0.15 and 0.65 mg kg^−1^ diet, respectively. Values are means ± SEM and ^a,b,c^ values with different superscripts were considered significant at *p* < .05.

### 3.2 LPS concentrations in colonic digesta and serum

**FIGURE 1:**
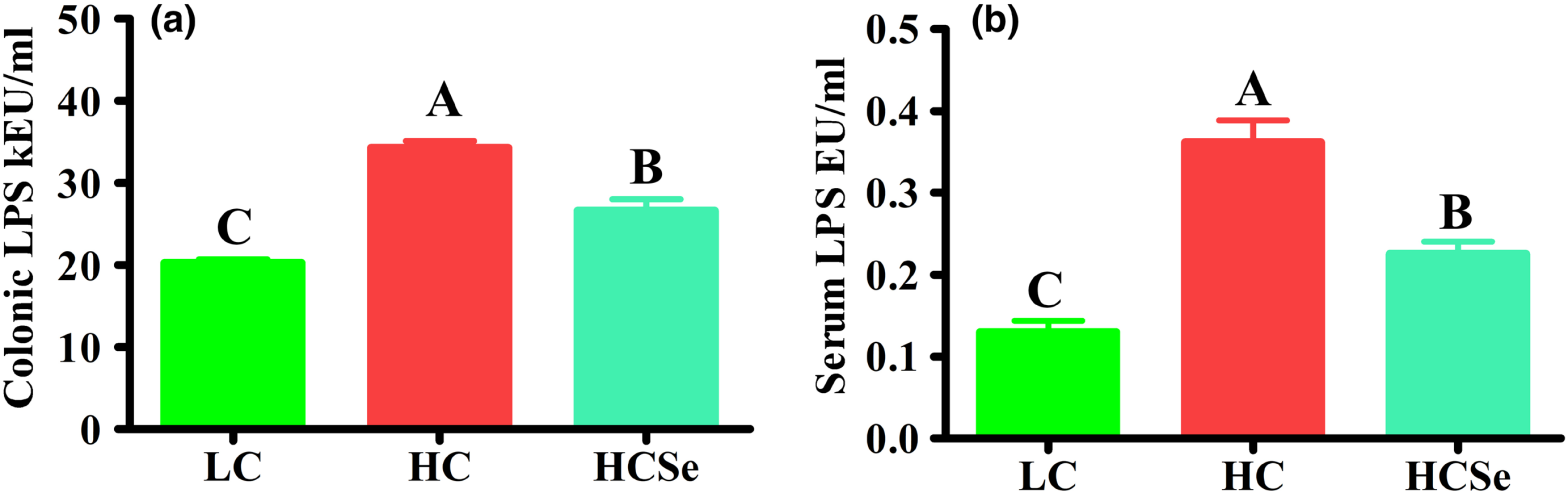
Effects of LC, HC, and HCSe diets on LPS contents in colonic digesta (a) and blood serum (b). The level of LPS contents was calculated as endotoxin unit per ml (EU/ml). Goats were fed low concentrate (LC), high concentrate (HC), and HC plus selenium (HCSe) diets for a period of 10 weeks. Total Se concentrations in LC, HC, and HCSe diets were 0.15, 0.15, and 0.65 mg kg^−1^ diet, respectively. Values are means **±** S.E and ^A,B,C^ different letters on the bars exhibit the difference among groups with *p* < .05

Lipopolysaccharides (LPS) concentrations increased (*p* < .001) by 69.12% and 179% (*p* < .05) in HC and HCSe goats, respectively than in LC goats. However, the Se‐supplemented (HCSe) diet caused significant reduction in HC‐induced increase of LPS concentrations by 22.35% (*p* < .001) in colonic digesta, and 37.68% (*p* < .005) in serum (Figure 1a and b).

### 3.3 Liver histology

**FIGURE 2:**
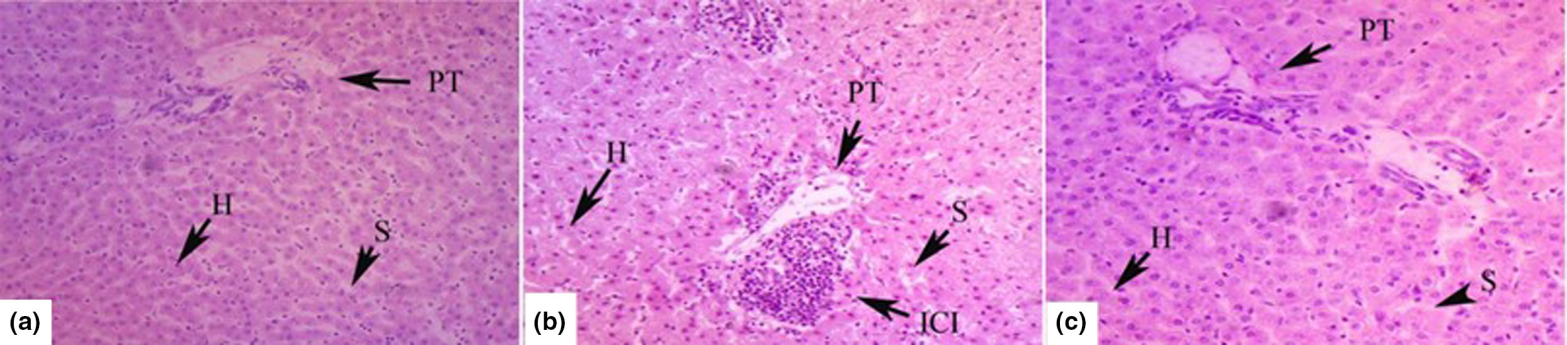
Representative microphotographs with hematoxylin and eosin staining. Comparison of histological changes in liver of LC (a), HC (b), and HCSe (c). Inflammatory cell infiltration (ICI) sinusoids (S), Hepatocytes (H), portal tract (PT). Goats were fed low concentrate (LC), high concentrate (HC) and HC plus selenium (HCSe) diets for a period of 10 weeks. Total Se concentrations in LC, HC, and HCSe diets were 0.15, 0.15, and 0.65 mg kg^−1^ diet, respectively

Goats fed LC diet exhibited normal liver histology with hepatic triads, each consisting of portal vein, arterial branch along with bile duct and cords of hepatocytes separated by sinusoids radiating from central venule (Figure 2a). However, HC diet induced pathological lesions such as severe inflammatory cell infiltration around portal area and mild dilation in sinusoids (Figure 2b). Whereas the Se addition ameliorated the HC diet‐induced abnormal histological changes in liver of HCSe goats (Figure 2c).

### 3.4 Oxidative stress markers in serum and liver

**FIGURE 3:**
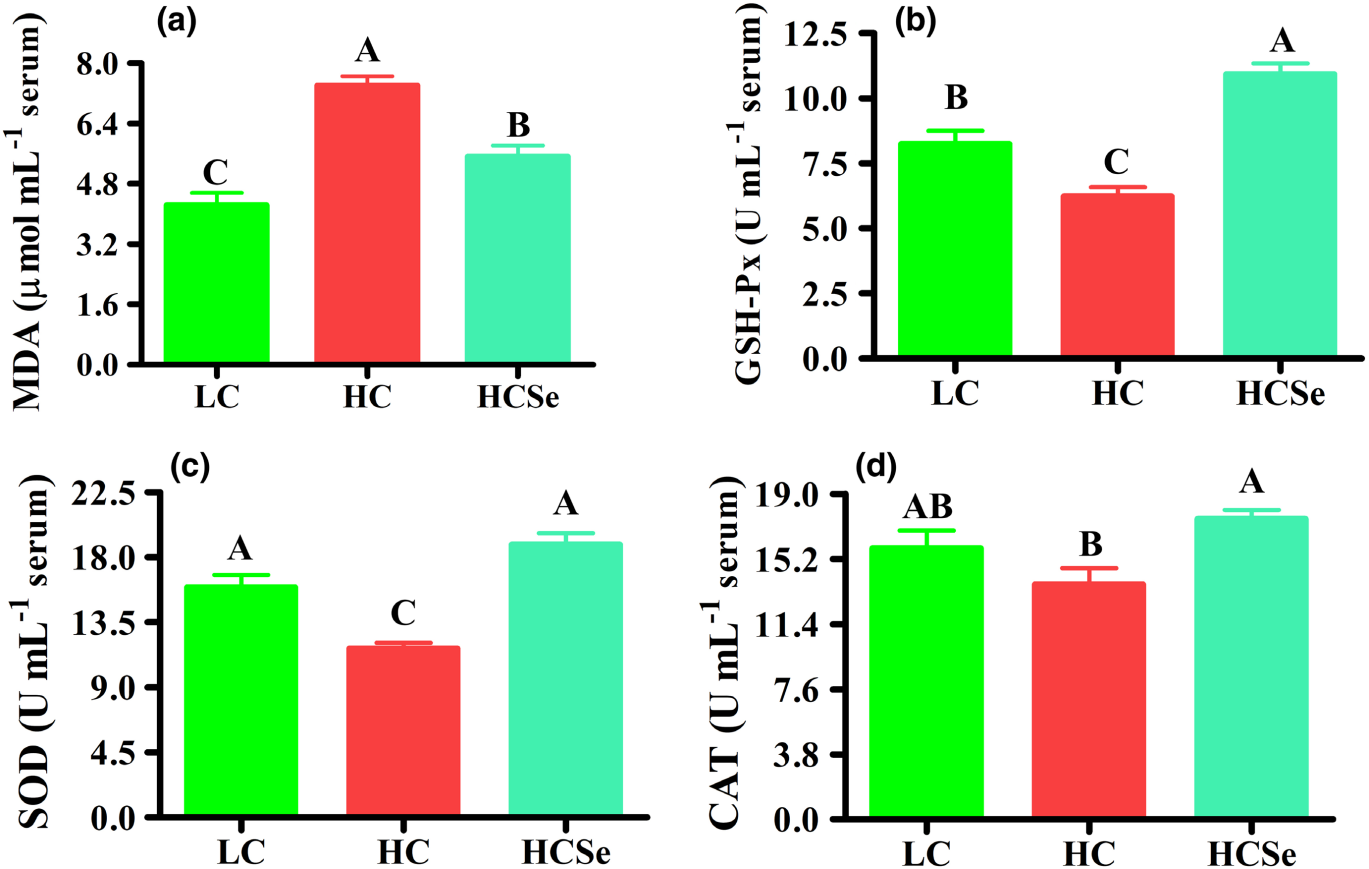
The effects of LC, HC, and HCSe diets on serum oxidative markers in goats; malondialdehyde (MDA) levels (a), and activities of glutathione peroxidase (GSH‐Px, b), superoxide dismutase (SOD, c) and catalase (CAT, d). Goats were fed low concentrate (LC), high concentrate (HC), and HC plus selenium (HCSe) diets for a period of 10 weeks. Total Se concentrations in LC, HC, and HCSe diets were 0.15, 0.15, and 0.65 mg kg^−1^ diet, respectively. Values are means ± S.E and ^A,B,C^ different letters on the bars exhibit the difference among groups with *p* < .05

**FIGURE 4:**
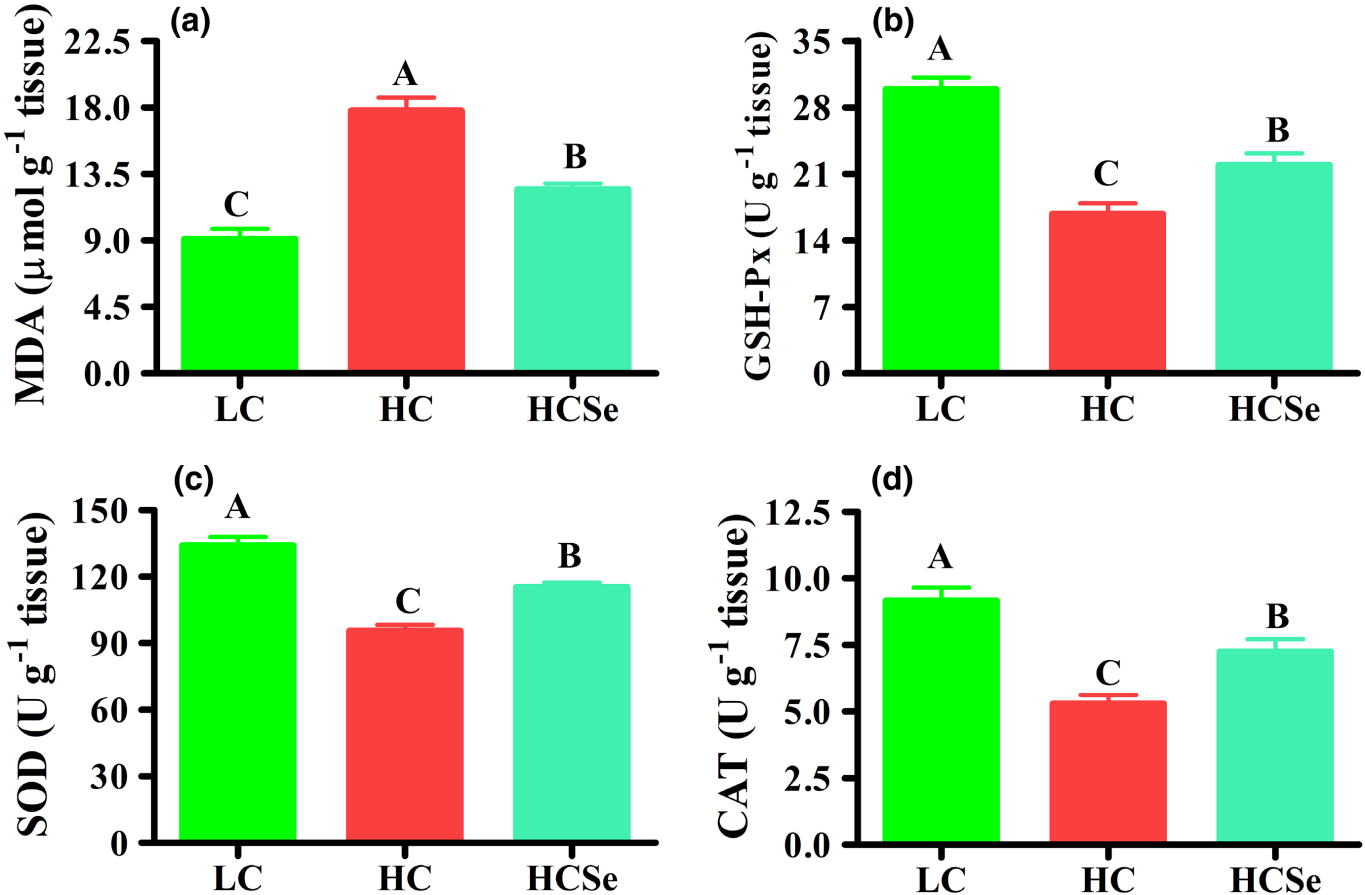
The effects of LC, HC, and HCSe diets on hepatic tissue oxidative markers in goats; malondialdehyde (MDA) levels (a), and activities of glutathione peroxidase (GSH‐Px, b), superoxide dismutase (SOD, c), and catalase (CAT, d). Goats were fed low concentrate (LC), high concentrate (HC), and HC plus selenium (HCSe) diets for a period of 10 weeks. Total Se concentrations in LC, HC, and HCSe diets were 0.15, 0.15, and 0.65 mg kg^−1^ diet, respectively. Values are means ± S.E and ^A,B,C^ different letters on the bars exhibit the difference among groups with *p* < .05

Assessment of oxidative stress markers (OS) showed that HC diet induced severe oxidative stress in serum and liver as reflected by highly significant increase (*p* < .001) in malondialdehyde (MDA) content by 75.16% and 95.28%, respectively in HC compared with LC goats. Whereas Se addition reduced OS by lowering MDA content (*p* < .01) in serum and liver by 25.52% and 29.94%, respectively in HCSe compared with HC goats (Figures 3a & 4a). Concurrent with increase in MDA content, the activities of antioxidant enzymes were significantly altered by different dietary treatments. Serum activities of glutathione peroxidase (GSH‐Px) and superoxide dismutase (SOD) were lowered (*p* < .05) by 24.4% and 26.59%, respectively in HC compared with LC goats, whereas increased (*p* < .001) by 75.34% and 61.39%, respectively in HCSe compared with HC group. However, serum SOD activity did not vary (*p* > .05) between LC and HCSe groups. Moreover, serum catalase (CAT) activity was not different (*p* > .05) between LC and HC diets. Nevertheless, Se‐added (HCSe) diet raised (*p* < .05) CAT activity by 28.03% in comparison with HC diet (Figure 3b–d).

The hepatic GSH‐Px, SOD, and CAT activities were declined (*p* < .001) by 43.95%, 28.76%, and 42.25%, respectively in HC compared with LC (Figure 3b–d), whereas increased by 23.58%, 17.23%, and 26.97%, respectively in Se‐fed (HCSe) compared with HC goats (Figure 4b–d).

### 3.5 Expression of inflammation‐related genes in liver

**FIGURE 5:**
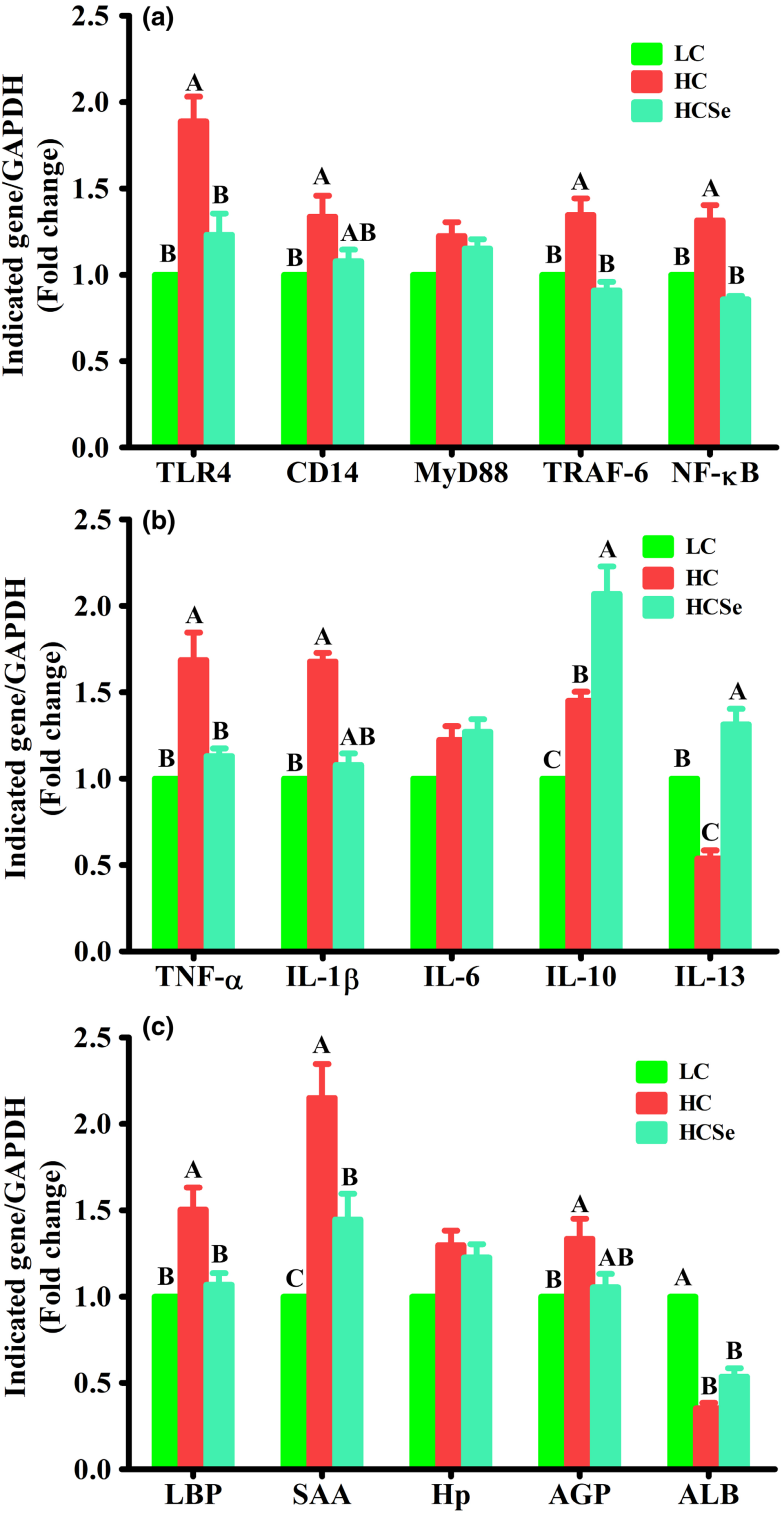
The effects of LC, HC, and HCSe diets on expression of immuno‐ inflammatory genes in liver of goats; transcriptional factors (a), cytokines (b), and acute phase proteins (c). Toll‐like receptor (TLR)‐4, cluster of differentiation (CD)‐14, myeloid differentiation (MyDD)‐88, tumor necrosis factor (TNF) ‐α, TNF receptor‐associated factor (TRAF)‐6, nuclear factor kappa light chain enhancer of activated B cells (NF‐κ B), Interluekins (IL)‐1β, IL‐6, IL‐10, IL‐13, Lipopolysachharide‐binding protein (LBP), Serum amyloid A (SAA), α‐Acid glycoprotein (AGP), Haptoglobin (Hp), Albumin (ALB), and Glyceraldehyde 3‐phosphate dehydrogenase (GAPDH). Goats were fed low concentrate (LC), high concentrate (HC), and HC plus selenium (HCSe) diets for a period of 10 weeks. Total Se concentrations in LC, HC, and HCSe diets were 0.15, 0.15, and 0.65 mg kg^−1^ diet, respectively. Values are means ± S.E and ^A,B,C^ different letters on the bars exhibit the difference among groups with *p* < .05

The mRNA expressions of immunoinflammatory genes including transcriptional factors, cytokines, and acute phase proteins in liver are shown in Figure 5. Compared with LC, the HC diet upregulated (*p* < .001 to <.05) the expressions of toll‐like receptor‐4 (TLR‐4), cluster of differentiation‐14 (CD‐14), tumor necrosis factor receptor‐associated factor‐6 (TRAF‐6), nuclear factor kappa B (NF‐κB). Addition of Se in diet reduced (*p* < .001) TLR4, TRAF6, and NF‐kB expressions by 34.75%, 32.5%, and 34.8%, respectively in HCSe compared with HC goats; however, CD‐14 expression was not significantly different (*p* > .05) between two groups. In addition, expressions of these genes did not differ (*p* > .05) between LC and HCSe goats. Moreover, Myeloid differentiation‐88 (MyDD‐88) mRNA expressions showed no difference (*p* > .05) among the three groups (Figure 5a).

The hepatic mRNA expression levels of pro‐inflammatory cytokines including tumor necrosis factor‐α (TNF‐α) and interleukin‐β (IL‐β) significantly increased (*p* < .001) by 1.55‐fold and 1.68‐fold, respectively, and that of anti‐inflammatory cytokine IL‐13 decreased (*p* < .001) by 0.52‐fold in HC compared with LC goats. In comparison with HC, Se supplementation downregulated (*p* < .001) hepatic TNF‐α and IL‐β expressions by 26.78% and 35.64%, respectively, and upregulated (*p* < .001) the IL‐13 expressions by 59.4% in HCSe goats. However, TNF‐α and IL‐β expressions did not vary (*p* > .05) between LC and HCSe groups. Compared with LC, the hepatic IL‐10 mRNA expression levels increased (*p* < .01) in both HC and HCSe goats by 1.45‐fold and 2.07‐fold, respectively; however, increase in IL‐10 expression was 42.7% higher in HCSe than in HC. Moreover, IL‐6 mRNA expressions were not different (*p* > .05) among three groups (Figure 5b).

The hepatic mRNA expression levels of positive acute phase proteins (APP) including lipopolysachharide‐binding protein (LBP), serum amyloid‐A (SAA), and α‐acid glycoprotein (AGP) were upregulated (*p* < .001 to <.05) by 1.5‐fold, 2.15‐fold, and 1.5‐fold, respectively, and the negative APP including albumin (ALB) downregulated (*p* < .001) by 0.65‐fold in HC compared with LC goats. In Se‐supplemented (HCSe) goats, the expressions of LBP, and SAA were lowered (*p* < .01) by 29% and 32.7%, respectively, compared with HC goats; however, the expressions of AGP and ALB were not different (*p* > .05) between HC and HCSe goats. Furthermore, SAA expression was 1.5‐folds higher (*p* < .01) and ALB expression was 0.46‐fold lower (*p* < .01) in HCSe compared with LC; however, the expressions of LBP, and AGP were not different (*p* > .05) between two groups. Hepatic haptoglobin (Hp) mRNA expressions showed no differences (*p* > .05) among three groups (Figure 5c).

## 4 DISCUSSION

### 4.1 Colonic fermentation pattern and LPS levels

It is very well established that feeding high concentrate (HC) diets enhance fermentation rate which causes an increase in concentrations of fermentative acids with simultaneous decrease in digesta pH in rumen and hindgut of mammals (Tao et al., 2017; Wang et al., 2018). In addition, the linear relationship between rise in fermentation rate with duration of HC diet intake indicates that prolonged HC feeding adversely affects the gut fermentation process (Wang et al., 2018). Similarly, feeding goats with HC diets for 10 weeks in our study elevated the concentrations of individual short chain fatty acid (SCFA), that is, acetate, propionate, and butyrate as well as the total SCFA (tSCFA) accompanied with lowered pH in colonic fluid compared with low concentrate (LC) diet‐fed goats. The increase in acetate concentration and acetate propionate ratio by 49.21% and 20.35 along with reduced pH suggest that fermentation process was negatively affected in HC compared with LC goats (Huhtanen et al., 1993). However, Se addition improved fermentation in HCSe compared with HC goats, which is consistent with findings of Samo et al. (2020).

Lipopolysaccharides (LPS) an integral part of external surface layer of gram‐negative bacteria, are commonly called as endotoxins which possess both antigenic and immunogenic properties (Cao et al., 2006; Ciesielska et al., 2021). Long‐term HC feeding results in elevation of free luminal LPS levels throughout gastrointestinal tract (GIT) including ruminal fluid, cecal, and colonic digesta, which appear in blood as well as in feces of ruminants (Li et al., 2012; Plaizier et al., 2017). In our study, concomitant with enhanced fermentation rate, LPS levels increased in colon and serum of HC goats. Previous studies have shown that feeding 60–90% grain‐rich diets to goats caused substantial increase in colonic and blood LPS levels (Tao et al., 2017; Wang, Peng, et al., 2021, Wang, Salem, et al., 2021). HC diet‐induced acidic luminal pH is believed to cause LPS production through bacteriolysis (Plaizier et al., 2017), and both the luminal acidity and LPS induce mucosal damages and cause LPS translocation through altered permeability of GIT wall (Abaker et al., 2017). Though the elevation of peripheral blood LPS levels may be caused by leakage from both rumen and hindgut epithelia, yet it is generally believed that the hind gut, because of being composed of monolayered epithelium and easily compromised by abnormal luminal environment, is a major site of translocation (Li et al., 2012). Several studies have shown increased starch flow and elevated amylase activity in digesta accompanied with severe epithelial injuries in cecum and colon of ruminants fed HC diets (Samo et al., 2020; Tao et al., 2017; Wang, Peng, et al., 2021; Wang, Salem, et al., 2021). Though LPS levels raised in both HC and HCSe goats, but LPS levels were significantly lower by *22*.35% and 37.68% in colon and serum of HCSe goats, respectively. Similar findings have been reported by previous studies in goats (Samo et al., 2020). However, the exact mechanism of lowering LPS levels by Se supplementation is not known. This might be partly due to reduced luminal‐free LPS release by inhibiting bacteriolysis and lowered translocation from GIT since Se supplementation has been shown to inhibit the LPS production and exert epithelial protective effects in colon of goats under concentrate‐diet stress (Samo et al., 2020) and in jejunum of pigs under heat stress (He et al. 2021). The inhibition of bacteriolysis might be due to increased antioxidant protection by Se incorporation in microbes (Čobanová et al., 2017). Moreover, the incorporation and retention of Se in microflora is considerably higher from organic than inorganic selenocompounds, and when supplemented with grain‐rich diets compared with low concentrate or forage diets (Koenig et al., 1997; Mainville et al., 2009).

### 4.2 Liver histopathology and oxidative stress

Concurrent with elevated serum LPS levels, HC diet induced pathological lesions characterized by severe inflammatory cell infiltration around portal area and mild dilation in sinusoids in liver. On leakage from GIT epithelium, the LPS enters the luminal veins and via the hepatic portal vein (PV) transported to liver, where exceeding the detoxification limits, the LPS damaging the hepatocytes escapes via hepatic vein (HV) into the peripheral circulation (Chang, Zhang, Xu, Jin, Seyfert, et al., 2015; Dong et al., 2013). Feeding HC diets reduced removal rate thereby retained LPS and induced inflammatory lesions and impairment in hepatocytes and raised peripheral blood LPS levels in PV, HV, and artery and jugular veins of goats and cattle (Chang, Zhang, Xu, Jin, Seyfert, et al., 2015; Dong et al., 2013; Guo et al., 2017).

LPS‐induced hepatic pathological lesions in HC diet‐fed animals are associated with oxidative stress (OS) which occurs because of an imbalance between the pro‐ and the antioxidants (Abaker et al., 2017; Duanmu et al., 2016). The amount of malondialdehyde (MDA) content represents the rate of lipid peroxidation and thus used as principal OS biomarker (Wang, Peng, et al., 2021, Wang, Salem, et al., 2021). The increasing amounts of reactive oxygen species (ROS) are immediately encountered by first‐line antioxidation defense system comprising of endogenous antioxidant (AO)‐enzymes including glutathione peroxidase (GSH‐Px), superoxide dismutase (SOD), and catalase (CAT) (Samo et al., 2020). In the present study, HC diet feeding induced OS as indicated by elevated MDA content accompanied with lowered AO‐enzymes activities in serum and hepatic tissues. Consistent with our results, previous studies have reported elevated MDA content, lowered activities of AO‐enzymes, and declined total antioxidant capacity (T‐AOC) liver and plasma, accompanied with modulation of SOD, GSH‐Px, and CAT genes expression in livers of goats and cattle fed HC diets (Abaker et al., 2017; Duanmu et al., 2016; Wang, Peng, et al., 2021; Wang, Salem, et al., 2021). Further studies at molecular level reported the hepatic downregulation of the nuclear factor erythroid2‐related factor 2 (NRF2) and total glucocorticoid receptor (GR) associated with higher GR nuclear translocation suggested that HC diet induced hepatic OS through LPS‐mediated suppression of NRF2‐dependent antioxidant signaling pathway and increased nuclear translocation of GR in ruminants' liver (Abaker et al., 2017; Dong et al., 2013; Wang, Peng, et al., 2021; Wang, Salem, et al., 2021).

However, the extra‐Se supplementation exerted hepatoprotective effects through mitigation of HC diet‐induced OS and pathological lesions in liver, which may be at least in part due to lowered release, translocation and thus entry of LPS into liver as explained in earlier section. Dietary Se supplementation ameliorated the hepatic OS by regulating the expressions of antioxidant genes via stimulating the Nrf2‐signaling pathway in pigs exposed to drug‐induced OS (Liu, Chen, et al., 2021). Besides, extra‐Se supplementation reduced LPS levels and attenuated oxidative injury goat colon (Samo et al., 2020) and improved blood antioxidant status in sheep and pigs under heat stress (Chen et al., 2020; Liu, Tang, et al., 2021; Mousaie, 2021). Moreover, the synthesis of selenoproteins such as GSH‐Px particularly in Se specific tissues like liver is associated with Se availability which consecutively depends upon dietary Se level (Čobanová et al., 2017). In the present study, although the Se level was five‐fold higher, the hepatic GSH‐Px activity was 26.66% lower in HCSe compared with LC goats; however, the serum GSH‐Px activity was 24.56% higher in HCSe goats. These results indicate the higher Se demand during HC diet feeding due to increasing utilization of Se by liver, not only for production of AO‐enzymes but also for synthesis and maintenance of other selenoproteins metabolism and to fulfill Se requirement of other tissues through maintenance of Se homeostasis (selenostasis). Moreover, HC diet‐induced pathological lesions are believed to be mediated via LPS, since the LPS‐exposure reduced Se concentrations and GSH‐Px activity, induced oxidative damage and impaired metabolism in livers of mice (Sherlock et al., 2020), sheep (Cao et al., 2006) and goats (Wang et al., 2017).

### 4.3 Liver inflammation

In the present study, concurrent with elevated serum LPS levels and hepatic oxidative injury, HC diet altered the expression of immunoinflammatory genes in liver. HC diet‐derived LPS elicits acute phase response (APR) by regulating the synthesis and secretion of acute phase proteins (APPs) in liver thus any fluctuation in their concentrations in the peripheral blood reflects the functional disturbance and inflammation of liver (Guo et al., 2017; He et al., 2019; Minuti et al., 2015). Many studies have reported that HC diets increased levels of positive APPs and decreased negative APPs in peripheral blood of ruminants (Chandra et al., 2018; Ohtaki et al., 2020; Zhang et al., 2016) accompanied with alteration at transcriptional level in liver (Chishti et al., 2020; Li et al., 2019). Moreover, the modulation of hepatic APPs genes expressions in LPS‐challenged goats, sheep, and cattle (Cao et al., 2006; Jiang et al., 2008; Wang et al., 2017) indicate that HC diet induce hepatic inflammatory response through LPS translocated from GIT via portal vein (Chang, Zhang, Xu, Jin, Guo, et al., 2015; Chang, Zhang, Xu, Jin, Seyfert, et al., 2015). Consistent with these data, we found higher mRNA expression levels of some positive APPs including LPS‐binding protein (LBP), serum amyloid A (SAA), and haptoglobin (Hp) and lower expression of a negative APP, albumin (ALB) in the liver of goats in HC than in LC group.

The first step in initiation of LPS‐induced inflammatory response is recognition and binding of LPS with LBP. A 60‐kDa protein, LBP is highly sensitive to LPS, and its plasma levels drastically raised up to 200% in goats fed HC diets and hence considered as reliable biomarker of systemic inflammation (Chang, Zhang, Xu, Jin, Seyfert, et al., 2015; Dong et al., 2013). SAA is the most sensitive AAP and frequently found upregulated in ruminants in response to variety of stresses, such as infections, trauma, and concentrated diets (Bochniarz et al., 2020; Jia et al., 2014), hence regarded as classical stress marker in ruminants. Previous studies have shown variable reports on hepatic Hp mRNA expression in ruminants fed concentrated diets. The hepatic Hp mRNA expression increased in lactating goats fed high grain diets for 8–9 weeks (Chang, Zhang, Xu, Jin, Guo, et al., 2015; Dong et al., 2013), whereas increasing feeding trial up to 12 weeks exhibited no significant change in Hp expression compared with goats fed low grain diets (Duanmu et al., 2016). Besides, Li et al. (2019) observed hepatic Hp upregulation in the newborn calves induced with subacute ruminal acidosis (SARA) through starch‐rich diet for 16 weeks, while Hp expression was not affected in adult dairy cows fed HC diet for 8 weeks (Guo et al., 2017). The upregulation of Hp is linked with ruminal acidosis, damaged GIT wall and elevated blood LPS levels thus the overexpression of Hp has been considered as potential marker of severe ruminal acidosis in ruminants (Girardi et al., 2018). Hence, lack of effect of HC diet on Hp expression suggests gradual adaptation to concentrated diet over a period (Chishti et al., 2020).

Plasma AGP level initially decreased and then subsequently increased in sheep fed HC diet (Girardi et al., 2018) and the LPS challenge increased hepatic AGP expression in cow (Jiang et al., 2008). Though the role of AGP is not fully understood but several studies have shown its protective effects against inflammation and thus considered as powerful anti‐inflammatory agent (Brown et al., 2021). In an in vitro study, it has been reported that AGP prevented hemoglobin‐induced OS through CD163 upregulation mediated either by IL‐6 or IL‐10 through TLR‐4 pathway activation (Komori et al., 2012). In current study, the increased hepatic mRNA expressions of AGP, TLR‐4 accompanied by upregulation of anti‐inflammatory cytokine IL‐10 in HC goats suggest the anti‐inflammatory effects of AGP. Albumins represent 40–60% of total proteins in circulation and the decreased albumin concentrations is a consequence of reduced synthesis which may be due to increased utilization of amino acids for the synthesis of cytokines and APPs (Girardi et al., 2018; Tothova et al., 2014). In accordance with our findings, feeding HC diet downregulated ALB gene expression in sheep (Chishti et al., 2020). Supranutritional Se supplementation attenuated the expressions of HC diet‐induced APPs in liver. Consistently, the increasing Se above the recommended levels (0.6–0.7 mg kg^−1^ diet) in diet reduced peri‐ and postpartum stress by improved immune response through attenuation of APPs including SAA, Hp, and albumin in dairy cows (Gong & Xiao, 2021; Hall et al., 2014) and alleviated hepatic metabolic disorder by recovering the gene expression profile in pigs under chronic heat stress (Liu, Tang, et al., 2021).

The alteration in APPs levels in HC fed ruminants is found associated with changes in pro‐ and anti‐inflammatory cytokines levels in blood (Chang, Zhang, Xu, Jin, Seyfert, et al., 2015; Guo et al., 2017; Ohtaki et al., 2020). Concomitantly, we observed hepatic mRNA upregulation of TNF‐α, IL‐1β, IL‐10, and downregulation of IL‐13 in HC than in LC goats. APPs production is stimulated by HC diet‐derived LPS in liver through activation of NF‐kB‐mediated TNF‐α‐IL‐1/IL‐6 signaling pathway in immune cells mainly Kpuffer cells, the principal hepatic macrophagocytes (Ciesielska et al., 2021; Guo et al., 2017; Kany et al., 2019). Upon stimulation and entry inside the nucleus, NF‐κB binds to proximal promoter regions of number of immuno‐inflammatory genes and induce transcription of cytokines, chemokines, and other inflammatory mediators in hepatic immune cells (Chang, Zhang, Xu, Jin, Guo, et al., 2015; Chang, Zhuang, Seyfert, Zhang, Xu, et al., 2015). It has demonstrated that HC diet‐derived LPS induce upregulation of NF‐κB mRNA and protein expressions which modulate TNF‐α, IL‐1β, IL‐6, and IL‐10 expressions and consequently lead to altered AAPs production in liver of goats and cattle (Dong et al., 2013; Duanmu et al., 2016; Guo et al., 2017). IL‐10 and IL‐13 play crucial role as endogenous inflammatory regulators and have been shown to exert protective effects against ischemic‐induced liver injury in mice (Kato et al., 2003). It has been suggested that the TLR‐4/MyD88 signaling pathway is involved in the stimulation of anti‐inflammatory mediators, like IL‐10 aiding to terminate the inflammation (Ciesielska et al., 2021). Consistently, the hepatic IL‐10 mRNA expressions were increased in goats fed HC diet and both IL‐10 and IL‐13 expressions were highly upregulated in goats fed extra Se‐added diet indicating that Se hastened the recovery from hepatic inflammation. IL‐10 expressions pattern seems to be related with period of HC diet treatment in the ruminants. Chang, Zhang, Xu, Jin, Guo, et al. (2015), Chang, Zhang, Xu, Jin, Seyfert, et al. (2015) found non‐significant but two‐fold increase in hepatic IL‐10 mRNA expression in goats fed HC diet for 6 weeks and its expression significantly increased by five‐fold when the dietary trial was extended up to 8 weeks. Similarly, rumen epithelial IL‐10 expressions were increased by two‐fold and five‐fold in goats fed HC diets for 6–8 weeks and 19 weeks, respectively (Hua et al., 2017; Zhang et al., 2017). This indicates that the gradual IL‐10 upregulation reduces inflammation and leads to adaptation to HC diet by the time. In addition, the intramammary LPS infusion induced its expression in the liver of cow (Jiang et al., 2008) indicating the contribution of HC diet‐derived LPS in elevated hepatic IL‐10 expression in this study. Conversely, Se supplementation increased hepatic IL‐10 expression in dose‐dependent manner with simultaneous reduction of inflammation and apoptosis in mice under acute alcoholism and the hepatoprotective effects were more pronounced with extra‐Se feeding (Zhang et al., 2017).

The activation and nuclear translocation of NF‐κB in target hepatocytes is stimulated by TLR4 signal transduction pathway. Once identified and bound to LBP, the LPS is transported to and via CD14 mediation delivered to TLR4/MD‐2 complex. This interaction activates TLR‐4 through recruitment of MyD88 on its intracellular fragment, which via TRAF‐6 signaling cascade stimulates NF‐κB and translocases in the nucleus (Ciesielska et al., 2021; Kany et al., 2019). In our findings, HC diet feeding increased the mRNA expressions of LBP, TLR‐4, CD14, and TRAF‐6 genes in liver. Though the expression of MyD88 genes was slightly higher but did not change significantly compared with LC goats. Similar findings have been reported in liver of goats fed grain‐rich diet (Dong et al., 2013; Duanmu et al., 2016). Moreover, the TLR‐4 upregulation was associated with lowered chromatin compaction and higher demethylation of TLR‐4 promoter (Chang, Zhang, Xu, Jin, Guo, et al., 2015; Chang, Zhuang, Seyfert, Zhang, Xu, et al., 2015) which suggests that HC diet‐induced epigenetic mechanism is involved in the activation of TLR‐4 pathway via LPS. TRAF‐6 is an important adapter protein which mediates the transduction of TLR‐4/Myd88 signals from cytoplasm to DNA inside the nucleus through NF‐κB activation where it induces cytokines‐encoding genes (Ciesielska et al., 2021; Kany et al., 2019). Guo et al. (2017) observed higher mRNA expressions of NF‐κB and TRAF‐6 genes accompanied by higher protein expression of NF‐κB in hepatic tissues of HC fed cows suggesting that HC diet‐derived LPS induce inflammatory response through NF‐κB‐mediated TLR‐4‐MyD88 pathway. Since the intramammary LPS infusion has been shown to upregulate the mRNA expressions of MyD88, P65 subunit of NF‐κB complex, TNF‐α, IL‐1β, and IL‐6 genes in the liver of cow (Jiang et al., 2008).

Conversely, extra‐Se supplementation in our study subsided hepatic inflammatory response by attenuation of LBP, TRAF‐6, NF‐κB, and TLR‐4 gene expression. Previous studies have shown that Se supplementation suppressed inflammation through TLR‐4 pathway regulation in liver, thymus, and jejunum of heat‐stressed and drug‐challenged growing pigs (He et al. 2021; Liu, Chen, et al., 2021) and in liver and uterus of LPS‐challenged rats and chicken (Al‐Dossari et al., 2020; Chen et al., 2020; Qu et al., 2020). Recent studies on the underlying molecular mechanism of Se immunomodulatory action against LPS‐induced endometritis in rats have revealed that Se hampered TLR‐4 migration to lipid rafts by bringing modification in lipid rafts through cholesterol depletion via LxRα‐ABCA1 pathway regulation (Chen et al., 2020). The present results demonstrated that high concentrate (HC) diet elevated colonic and blood LPS levels and induced histopathological changes associated with oxidative stress and inflammation in liver. Moreover, HC diet‐induced hepatic inflammation was associated with NF‐kB‐TNF‐α‐IL‐1/IL‐6‐mediated TLR‐4 signaling pathway. However, the supranutritional Se level attenuated HC diet‐induced oxidative stress and inflammation and thus diminished the liver injury in goats.

## CONFLICT OF INTEREST

The authors declare no conflict of interest.

## References

[fsn32980-bib-0001] Abaker, J. , Xu, T. , Jin, D. , Chang, G. , Zhang, K. , & Shen, X. (2017). Lipopolysaccharide derived from the digestive tract provokes oxidative stress in the liver of dairy cows fed a high‐grain diet. Journal of Dairy Science, 100(1), 666–678. 10.3168/jds.2016-10871 27865500

[fsn32980-bib-0002] Ahmed, Z. , Malhi, M. , Soomro, S. A. , Gandahi, J. A. , Arijo, A. , Bhutto, B. , & Qureshi, T. (2016). Dietary selenium yeast supplementation improved some villi morphological characteristics in duodenum and jejunum of young goats. Journal of Animal and Plant Science, 26(2), 382–387.

[fsn32980-bib-0003] Al‐Dossari, M. H. , Fadda, L. M. , Attia, H. A. , Hasan, I. H. , & Mahmoud, A. M. (2020). Curcumin and selenium prevent lipopolysaccharide/diclofenac‐induced liver injury by suppressing inflammation and oxidative stress. Biological Trace Element Research, 196(1), 173–183. 10.1007/s12011-019-01910-4 31654258

[fsn32980-bib-0004] Bano, I. , Malhi, M. , Khatri, P. , Soomro, S. A. , Sajjad, H. , Leghari, A. , Awais, M. , Kandhro, S. , Lakho, S. A. , & Soomro, M. (2019). Effect of dietary selenium yeast supplementation on morphology and antioxidant status in testes of young goat. Pakistan Journal of Zoology, 51(3), 979. 10.17582/journal.pjz/2019.51.3.979.988

[fsn32980-bib-0005] Bochniarz, M. , Szczubiał, M. , Brodzki, P. , Krakowski, L. , & Dąbrowski, R. (2020). Serum amyloid A as an marker of cow֨ s mastitis caused by Streptococcus sp. Comparative Immunology, Microbiology and Infectious Diseases, 72, 101498. 10.1016/j.cimid.2020.101498 32505957

[fsn32980-bib-0006] Brown, W. , Garcia, M. , Mamedova, L. , Christman, K. , Zenobi, M. , Staples, C. , Leno, B. M. , Overton, T. R. , Whitlock, B. K. , Daniel, J. A. , & Bradford, B. J. (2021). Acute‐phase protein α‐1‐acid glycoprotein is negatively associated with feed intake in postpartum dairy cows. Journal of Dairy Science, 104(1), 806–817. 10.3168/jds.2020-19025 33131805

[fsn32980-bib-0007] Cao, H. , Kabaroff, L. C. , You, Q. , Rodriguez, A. , Boermans, H. J. , & Karrow, N. A. (2006). Characterization of ovine hepatic gene expression profiles in response to *Escherichia coli* lipopolysaccharide using a bovine cDNA microarray. BMC Veterinary Research, 2(1), 1–7. 10.1186/1746-6148-2-34 17134499 PMC1684251

[fsn32980-bib-0008] Chandra, R. A. , Wang, Y. , Zhang, H. , Roy, S. , Dai, H. , Chang, G. , & Shen, X. (2018). Sodium butyrate mitigates iE‐DAP induced inflammation caused by high‐concentrate feeding in liver of dairy goats. Journal of Agricultural and Food Chemistry, 66(34), 8999–9009. 10.1021/acs.jafc.8b02732 30078321

[fsn32980-bib-0009] Chang, G. , Zhang, K. , Xu, T. , Jin, D. , Guo, J. , Zhuang, S. , & Shen, X. (2015). Epigenetic mechanisms contribute to the expression of immune related genes in the livers of dairy cows fed a high concentrate diet. PLoS One, 10(4), e0123942. 10.1371/journal.pone.0123942 25860644 PMC4393131

[fsn32980-bib-0010] Chang, G. , Zhang, K. , Xu, T. , Jin, D. , Seyfert, H. M. , Shen, X. , & Zhuang, S. (2015). Feeding a high‐grain diet reduces the percentage of LPS clearance and enhances immune gene expression in goat liver. BMC Veterinary Research, 11(1), 1–11. 10.1186/s12917-015-0376-y 25889631 PMC4414381

[fsn32980-bib-0011] Chang, G. , Zhuang, S. , Seyfert, H. M. , Zhang, K. , Xu, T. , Jin, D. , Guo, J. , & Shen, X. (2015). Hepatic TLR4 signaling is activated by LPS from digestive tract during SARA, and epigenetic mechanisms contribute to enforced TLR4 expression. Oncotarget, 6(36), 38578–38590. 10.18632/oncotarget.6161 26498350 PMC4770722

[fsn32980-bib-0012] Chen, Y. , Zhao, Y. F. , Yang, J. , Jing, H. Y. , Liang, W. , Chen, M.‐y. , Yang, M. , Wang, Y. , & Guo, M. Y. (2020). Selenium alleviates lipopolysaccharide‐induced endometritis via regulating the recruitment of TLR4 into lipid rafts in mice. Food Function, 11(1), 200–210. 10.1039/c9fo02415h 31845693

[fsn32980-bib-0013] Chishti, G. A. , Salfer, I. J. , Nedelkov, K. V. , & Felix, T. L. (2020). Impacts of time‐fed concentrate‐based diets on plasma metabolites, rumen histology, and mRNA expression of hepatic enzymes of wethers. Animals, 10(4), 686. 10.3390/ani10040686 PMC7222829 32326483

[fsn32980-bib-0014] Ciesielska, A. , Matyjek, M. , & Kwiatkowska, K. (2021). TLR4 and CD14 trafficking and its influence on LPS‐induced pro‐inflammatory signaling. Cellular and Molecular Life Sciences, 78(4), 1233–1261. 10.1007/s00018-020-03656-y 33057840 PMC7904555

[fsn32980-bib-0015] Čobanová, K. , Faix, Š. , Plachá, I. , Mihaliková, K. , Váradyová, Z. , Kišidayová, S. , & Grešáková, Ľ. (2017). Effects of different dietary selenium sources on antioxidant status and blood phagocytic activity in sheep. Biological Trace Element Research, 175(2), 339–346. 10.1007/s12011-016-0794-0 27411926

[fsn32980-bib-0016] Del‐Razo‐Rodriguez, O. , Ramirez‐Bribiesca, J. , Lopez‐Arellano, R. , Revilla‐Vazquez, A. , Gonzalez‐Munoz, S. , Cobos‐Peralta, M. A. , Hernandez‐Calva, L. M. , & McDowell, L. (2013). Effects of dietary level of selenium and grain on digestive metabolism in lambs. Czech Journal of Animal Science, 58(6), 253–261.

[fsn32980-bib-0017] Dong, H. , Wang, S. , Jia, Y. , Ni, Y. , Zhang, Y. , Zhuang, S. , Shen, X. , & Zhao, R. (2013). Long‐term effects of subacute ruminal acidosis (SARA) on milk quality and hepatic gene expression in lactating goats fed a high‐concentrate diet. PLoS One, 8(12), e82850. 10.1371/journal.pone.0082850 24376594 PMC3871605

[fsn32980-bib-0018] Duanmu, Y. , Cong, R. , Tao, S. , Tian, J. , Dong, H. , Zhang, Y. , Ni, Y. , & Zhao, R. (2016). Comparative proteomic analysis of the effects of high‐concentrate diet on the hepatic metabolism and inflammatory response in lactating dairy goats. Journal of Animal Science and Biotechnology, 7(1), 5–11. 10.1186/s40104-016-0065-0 26855776 PMC4744397

[fsn32980-bib-0019] Girardi, A. M. , Sabes, A. F. , Fagliari, J. J. , da Silva, P. C. , De Oliveira, J. A. , & Marques, L. C. (2018). Changes in the levels of acute‐phase protein and other serum protein fractions in Santa Inês ewes fed with a high‐concentrate diet. Small Ruminant Research, 162, 34–38. 10.1016/j.smallrumres.2018.03.001

[fsn32980-bib-0020] Gong, J. , & Xiao, M. (2021). Increasing selenium supply during the close‐up dry period improves nutrient metabolism and attenuates inflammatory response after calving in dairy cows. Animal Science Journal, 92(1), e13551. 10.1111/asj.13551 33847030

[fsn32980-bib-0021] Guo, J. , Chang, G. , Zhang, K. , Xu, L. , Jin, D. , Bilal, M. S. , & Shen, X. (2017). Rumen‐derived lipopolysaccharide provoked inflammatory injury in the liver of dairy cows fed a high‐concentrate diet. Oncotarget, 8(29), 46769–46780. 10.18632/oncotarget.18151 28596485 PMC5564522

[fsn32980-bib-0022] Hall, J. A. , Bobe, G. , Vorachek, W. R. , Kasper, K. , Traber, M. G. , Mosher, W. D. , Pirelli, G. J. , & Gamroth, M. (2014). Effect of supranutritional organic selenium supplementation on postpartum blood micronutrients, antioxidants, metabolites, and inflammation biomarkers in selenium‐replete dairy cows. Biological Trace Element Research, 161(3), 272–287. 10.1007/s12011-014-0107-4 25142062

[fsn32980-bib-0023] He, M. , Li, L. , Wang, H. , Yan, S. , & Zhang, Y. (2019). Effects of high‐grain diet with buffering agent on the hepatic metabolism in lactating goats. Frontiers in Physiology, 10, 661. 10.3389/fphys.2019.00661 31191354 PMC6548822

[fsn32980-bib-0024] He, Y. , Liu, Y. , Tang, J. , Jia, G. , Liu, G. , Tian, G. , Chen, X. , Cai, J. , Kang, B. , & Zhao, H. (2022). Selenium exerts protective effects against heat stress induced barrier disruption and inflammation response in jejunum of growing pigs. Journal of the Science of Food and Agriculture, 102(2), 496–504. 10.1002/jsfa.11377 34145905

[fsn32980-bib-0025] Hernández‐Calva, L. , Guerrero‐Legarreta, M. , Pérez‐Chabela, M. , López‐Arellano, R. , & Ramírez‐Bribiesca, J. (2007). Interaction of dietary selenium and magnesium level on digestive function in lambs fed high‐concentrate diets. Journal of Applied Animal Research, 31(1), 41–46. 10.1080/09712119.2007.9706627

[fsn32980-bib-0026] Hua, C. , Tian, J. , Tian, P. , Cong, R. , Luo, Y. , Geng, Y. , Tao, S. , Ni, Y. , & Zhao, R. (2017). Feeding a high concentration diet induces unhealthy alterations in the composition and metabolism of ruminal microbiota and host response in a goat model. Frontiers in Microbiology, 8, 138. 10.3389/fmicb.2017.00138 28210249 PMC5288341

[fsn32980-bib-0027] Huhtanen, P. , Miettinen, H. , & Ylinen, M. (1993). Effect of increasing ruminal butyrate on milk yield and blood constituents in dairy cows fed a grass silage‐based diet. Journal of Dairy Science, 76(4), 1114–1124. 10.3168/jds.S0022-0302(93)77440-8 8486840

[fsn32980-bib-0028] Jia, Y. , Wang, S. , Ni, Y. , Zhang, Y. , Zhuang, S. , & Shen, X. (2014). High concentrate‐induced subacute ruminal acidosis (SARA) increases plasma acute phase proteins (APPs) and cortisol in goats. Animal, 8(9), 1433–1438. 10.1017/S1751731114001128 24852750

[fsn32980-bib-0029] Jiang, L. , Sørensen, P. , Røntved, C. , Vels, L. , & Ingvartsen, K. L. (2008). Gene expression profiling of liver from dairy cows treated intra‐mammary with lipopolysaccharide. BMC Genomics, 9(1), 1–12. 10.1186/1471-2164-9-443 18816405 PMC2576255

[fsn32980-bib-0030] Kany, S. , Vollrath, J. T. , & Relja, B. (2019). Cytokines in inflammatory disease. International Journal of Molecular Sciences, 20(23), 6008. 10.3390/ijms20236008 PMC6929211 31795299

[fsn32980-bib-0031] Kato, A. , Okaya, T. , & Lentsch, A. B. (2003). Endogenous IL‐13 protects hepatocytes and vascular endothelial cells during ischemia/reperfusion injury. Hepatology, 37(2), 304–312. 10.1053/jhep.2003.50075 12540780

[fsn32980-bib-0032] Koenig, K. , Rode, L. , Cohen, R. , & Buckley, W. (1997). Effects of diet and chemical form of selenium on selenium metabolism in sheep. Journal of Animal Science, 75(3), 817–827. 10.2527/1997.753817x 9078502

[fsn32980-bib-0033] Komori, H. , Watanabe, H. , Shuto, T. , Kodama, A. , Maeda, H. , Watanabe, K. , Kai, H. , Otagiri, M. , & Maruyama, T. (2012). α1‐acid glycoprotein up‐regulates CD163 via TLR4/CD14 protein pathway: Possible protection against hemolysis‐induced oxidative stress. Journal of Biological Chemistry, 287(36), 30688–30700. 10.1074/jbc.M112.353771 22807450 PMC3436313

[fsn32980-bib-0034] Li, W. , Gelsinger, S. , Edwards, A. , Riehle, C. , & Koch, D. (2019). Transcriptome analysis of rumen epithelium and meta‐transcriptome analysis of rumen epimural microbial community in young calves with feed induced acidosis. Scietific Reports, 9, 1–13. 10.1038/s41598-019-40375-2 PMC6426933 30894588

[fsn32980-bib-0035] Li, S. , Khafipour, E. , Krause, D. O. , Kroeker, A. , Rodriguez‐Lecompte, J. C. , Gozho, G. N. , & Plaizier, J. C. (2012). Effects of subacute ruminal acidosis challenges on fermentation and endotoxins in the rumen and hindgut of dairy cows. Journal of Dairy Science, 95, 294–303. 10.3168/jds.2011-4447 22192209

[fsn32980-bib-0036] Liu, L. , Chen, D. , Yu, B. , Luo, Y. , Huang, Z. , Zheng, P. , Mao, X. , Yu, J. , Luo, J. , Yan, H. , & He, J. (2021). Influences of selenium‐enriched yeast on growth performance, immune function, and antioxidant capacity in weaned pigs exposure to oxidative stress. Biomed Res Int, 2021, 1–11. 10.1155/2021/5533210 33855070 PMC8019624

[fsn32980-bib-0037] Liu, Y. , Tang, J. , He, Y. , Jia, G. , Liu, G. , Tian, G. , Chen, X. , Cai, J. , Kang, B. , & Zhao, H. (2021). Selenogenome and AMPK signal insight into the protective effect of dietary selenium on chronic heat stress‐induced hepatic metabolic disorder in growing pigs. Journal of Animal Science and Biotechnology, 12(1), 68–14. 10.1186/s40104-021-00590-2 34116728 PMC8196429

[fsn32980-bib-0038] Mainville, A. , Odongo, N. , Bettger, W. , McBride, B. , & Osborne, V. (2009). Selenium uptake by ruminal microorganisms from organic and inorganic sources in dairy cows. Canadian Journal of Animal Science, 89(1), 105–110. 10.4141/CJAS08031

[fsn32980-bib-0039] Malhi, M. , Gui, H. , Yao, L. , Aschenbach, J. R. , Gäbel, G. , & Shen, Z. (2013). Increased papillae growth and enhanced short‐chain fatty acid absorption in the rumen of goats are associated with transient increases in cyclin D1 expression after ruminal butyrate infusion. Journal of Dairy Science, 96(12), 7603–7616. 10.3168/jds.2013-6700 24119813

[fsn32980-bib-0040] Minuti, A. , Palladino, A. , Khan, M. , Alqarni, S. , Agrawal, A. , Piccioli‐Capelli, F. , Hidalgo, F. , Cardoso, F. C. , Trevisi, E. , & Loor, J. J. (2015). Abundance of ruminal bacteria, epithelial gene expression, and systemic biomarkers of metabolism and inflammation are altered during the peripartal period in dairy cows. Journal of Dairy Science, 98(12), 8940–8951. 10.3168/jds.2015-9722 26409956

[fsn32980-bib-0041] Moolchand, M. , Wang, J. , Gui, H. , & Shen, Z. (2013). Ruminal butyrate infusion increased papillae size and digesta weight but did not change liquid flow rate in the rumen of goats. Journal of Animal and Plant Sciences, 23(6), 1516–1521.

[fsn32980-bib-0042] Mousaie, A. (2021). Dietary supranutritional supplementation of selenium‐enriched yeast improves feed efficiency and blood antioxidant status of growing lambs reared under warm environmental condition. Tropical Animal Health and Production, 53(1), 1–7. 10.1007/s11250-021-02588-4 33486618

[fsn32980-bib-0043] Ohtaki, T. , Ogata, K. , Kajikawa, H. , Sumiyoshi, T. , Asano, S. , Tsumagari, S. , & Horikita, T. (2020). Effect of high‐concentrate corn grain diet‐induced elevated ruminal lipopolysaccharide levels on dairy cow liver function. Journal of Veterinary Medical Science, 82(7), 971–977. 10.1292/jvms.20-0117 32461536 PMC7399309

[fsn32980-bib-0044] Plaizier, J. C. ; Li, S. ; Tun, H. M. ; & Khafipour, E. (2017). Nutritional models of experimentally‐induced subacute ruminal acidosis (SARA) differ in their impact on rumen and hindgut bacterial communities in dairy cows. Frontiers in Microbiology, 7, 2128. 10.3389/fmicb.2016.02128 28179895 10.3389/fmicb.2016.02128 PMC5265141

[fsn32980-bib-0045] Qu, J. , Wang, W. , Zhang, Q. , & Li, S. (2020). Inhibition of lipopolysaccharide‐induced inflammation of chicken liver tissue by selenomethionine via TLR4‐NF‐κB‐NLRP3 signaling pathway. Biological Trace Element Research, 195(1), 205–214. 10.1007/s12011-019-01841-0 31332706

[fsn32980-bib-0046] Samo, S. P. , Malhi, M. , Gadahi, J. A. , Lei, Y. , Kaciwal, A. B. , & Soomro, S. A. (2018). Effect of organic selenium supplementation in diet on gastrointestinal tract performance and meat quality of goat. Pakistan Journal of Zoology, 50(3), 995–1001. 10.17582/journal.pjz/2018.50.3.995.1001

[fsn32980-bib-0047] Samo, S. P. , Malhi, M. , Kachiwal, A. B. , Gadahi, J. A. , Parveen, F. , Kalhoro, N. H. , & Lei, Y. (2020). Supranutritional selenium level minimizes high concentrate diet‐induced epithelial injury by alleviating oxidative stress and apoptosis in colon of goat. BMC Veterinary Research, 16(1), 1–10. 10.1186/s12917-020-02653-4 33246474 PMC7694315

[fsn32980-bib-0048] Shahid, A. B. , Malhi, M. , Soomro, S. A. , Shah, M. G. , Kalhoro, N. H. , Kaka, A. , Mal, R. , Soomro, M. A. , Samo, S. , & Sanjrani, M. N. (2020). Influence of dietary selenium yeast supplementation on fermentation pattern, papillae morphology and antioxidant status in rumen of goat. Pakistan Journal of Zoology, 52(2), 565. 10.17582/journal.pjz/20190205120240

[fsn32980-bib-0049] Sherlock, L. G. , Kara‐Sjostrom, L. S. , Delaney, C. , Tipple, T. E. , Krebs, N. F. , Nozik‐Grayck, E. , & Wright, C. J. (2020). Hepatic‐specific decrease in the expression of selenoenzymes and factors essential for selenium processing after endotoxemia. Frontiers in Microbiology, 11, 1–12. 10.3389/fimmu.2020.595282 33224150 PMC7674557

[fsn32980-bib-0050] Tao, S. , Tian, P. , Luo, Y. , Tian, J. , Hua, C. , Geng, Y. , Cong, R. , Ni, Y. , & Zhao, R. (2017). Microbiome‐metabolome responses to a high‐grain diet associated with the hind‐gut health of goats. Frontiers in Microbiology, 8, 1764. 10.3389/fmicb.2017.01764 28959247 PMC5603706

[fsn32980-bib-0051] Taylor, J. (2005). Time‐dependent influence of supranutritional organically bound selenium on selenium accumulation in growing wether lambs. Journal of Animal Science, 83(5), 1186–1193. 10.2527/2005.8351186x 15827263

[fsn32980-bib-0052] Tothova, C. , Nagy, O. , & Kovac, G. (2014). Acute phase proteins and their use in the diagnosis of diseases in ruminants: A review. Veterinární Medicína, 59(4), 163–180. 10.17221/7478-vetmed

[fsn32980-bib-0053] Wang, B. , Jia, M. , Fang, L. , Jiang, L. , & Li, Y. (2018). Effects of eucalyptus oil and anise oil supplementation on rumen fermentation characteristics, methane emission, and digestibility in sheep. Journal of Animal Science, 96(8), 3460–3470. 10.1093/jas/sky216 29860505 PMC6095444

[fsn32980-bib-0054] Wang, L. , Jia, S. , Yang, G. , Liu, R. , Yang, G. , Li, M. , Zhu, H. S. , Wang, Y. Y. , & Han, L. Q. (2017). The effects of acute lipopolysaccharide challenge on dairy goat liver metabolism assessed with 1 HNMR metabonomics. Journal of Animal Physiology and Animal Nutrition, 101(1), 180–189. 10.1111/jpn.12439 26847913

[fsn32980-bib-0055] Wang, K. , Peng, X. , Lv, F. , Zheng, M. , Long, D. , Mao, H. , Si, H. , & Zhang, P. (2021). Microbiome‐metabolites analysis reveals unhealthy alterations in the gut microbiota but improved meat quality with a high‐rice diet challenge in a small ruminant model. Animals, 11(8), 2306. 10.3390/ani11082306 34438763 PMC8388442

[fsn32980-bib-0056] Wang, Y. , Salem, A. Z. , Tan, Z. , Kang, J. , & Wang, Z. (2021). Activation of glucocorticoid receptors is associated with the suppression of antioxidant responses in the liver of goats fed a high‐concentrate diet. Italian Journal of Animal Science, 20(1), 195–204. 10.1080/1828051X.2021.1873706

[fsn32980-bib-0057] Zhang, Z. , Guo, Y. , Qiu, C. , Deng, G. , & Guo, M. (2017). Protective action of Se‐supplement against acute alcoholism is regulated by Selenoprotein P (SelP) in the liver. Biological Trace Element Research, 175(2), 375–387. 10.1007/s12011-016-0780-6 27334433

[fsn32980-bib-0058] Zhang, R. , Liu, Y. , Yin, Y. , Jin, W. , Mao, S. , & Liu, J. (2019). Response of rumen microbiota, and metabolic profiles of rumen fluid, liver and serum of goats to high‐grain diets. Animal, 13(9), 1855–1864. 10.1017/S1751731118003671 30614430

[fsn32980-bib-0059] Zhang, K. , Meng, M. , Gao, L. , Tu, Y. , & Bai, Y. (2019). Rumen‐derived lipopolysaccharide induced ruminal epithelium barrier damage in goats fed a high‐concentrate diet. Microbial Pathogenesis, 131, 81–86. 10.1016/j.micpath.2019.02.007 30910720

[fsn32980-bib-0060] Zhang, R. , Zhu, W. , & Mao, S. (2016). High‐concentrate feeding upregulates the expression of inflammation‐related genes in the ruminal epithelium of dairy cattle. Journal of Animal Science and Biotechnology, 7(1), 1–13. 10.1186/s40104-016-0100-1 27478614 PMC4966727

